# Validation of the Test Method—Determination of Available Carbohydrates in Cereal and Cereal Products, Dairy Products, Vegetables, Fruit, and Related Food Products and Animal Feeds: Collaborative Study, Final Action 2020.07

**DOI:** 10.1093/jaoacint/qsac116

**Published:** 2022-09-30

**Authors:** Ciara McLoughlin, Vincent A McKie, Barry V McCleary

**Affiliations:** Megazyme, Ltd, Bray Business Park, Southern Cross Road, Bray, County Wicklow A98YV29, Ireland; Megazyme, Ltd, Bray Business Park, Southern Cross Road, Bray, County Wicklow A98YV29, Ireland; FiberCarb, Murrumburrah, Eden Road, Greystones A63YW01, Ireland

## Abstract

**Background:**

A simple, accurate, and reliable method to measure available carbohydrate components of food products, including cereal and dairy products, fruits, vegetables, processed food, food ingredients, and animal foods, was developed by Megazyme (product K-AVCHO, Bray, Ireland). A single-laboratory validation of the enzymatic method resulted in First Action status as *Official Method of Analysis*^SM^**2020.07**.

**Objective:**

A collaborative study was conducted to evaluate the repeatability and reproducibility of *Official Method* **2020.07** for the measurement of available carbohydrates, including digestible starch, lactose, sucrose, isomaltose, maltose, glucose, fructose, and galactose in a broad range of food and feed products.

**Method:**

Samples are defatted if containing >10% fat content, and incubated with pancreatic α-amylase and amyloglucosidase under conditions that simulate those in the small intestine (pH 6, 37°C, 4 h). The reaction solution is clarified and diluted, and an aliquot is incubated with sucrase, maltase, oligo-1,6-α-glucosidase, and β-galactosidase to hydrolyze sucrose, maltose, isomaltose, and lactose to glucose, fructose, and galactose, which are then measured enzymatically. The multi-laboratory validation (MLV) matrixes included cereal, animal feeds, fruit, vegetables, infant formula, powdered milk drink, a dessert product, and mushrooms. Additional materials were analyzed by collaborators as “practice samples.”

**Results:**

All MLV matrixes resulted in repeatability relative standard deviations (RSD_r_) <3.91% and reproducibility relative standard deviations (RSD_R_) ranging from 3.51 to 11.58% with 9 of the 10 matrixes having RSD_R_ of <6.19%. For the practice samples, the RSD_R_ ranged from 2.7 to 11.4% with 7 of the 8 samples having RSD_R_ of <4.4%.

**Conclusions:**

*Official Method* **2020.07** meets the AOAC requirements for repeatability and reproducibility, and the data support Final Action status.

**Highlights:**

*Official Method* **2020.07** is a robust, simple to use, and reproducible method for the analysis of available carbohydrates in a wide range of matrixes.

Available carbohydrates are carbohydrates that are digested in the human small intestine. A method for direct analysis of glucose, fructose, sucrose, lactose, maltose, and starch in foods was introduced by McCance and Lawrence in 1929 (1). The individual values were summed to give a measure of available carbohydrates. This has been the basis of available carbohydrate analysis in the UK since that time. A “by difference” method for determining total carbohydrates was introduced by Atwater and Woods in 1896 (2), and this procedure is in use in the United States and many other countries. In this method, the moisture, protein, fat, ash, and alcohol content of a food are determined and then subtracted from the total weight of the food, and the remainder (or difference) is total carbohydrate. “Net carbohydrates” is determined by subtracting dietary fiber from the total carbohydrate value. This net carbohydrate value is equated with “available carbohydrates” determined directly. However, the “by difference” figure includes noncarbohydrate components such as lignin, organic acids, tannins, waxes, and some Maillard products. Also, it combines all the analytical errors from the other analyses (3). The McCance and Lawrence method (1) for measurement of available carbohydrates is limited by the fact that total starch is measured, instead of digestible starch, and sucrose is measured after hydrolysis to glucose and fructose by invertase (β-fructofuranosidase), an enzyme that also hydrolyzes a low degree of polymerization (DP) fructo-oligosaccharides (FOS) (4). A method for the measurement of digestible starch and resistant starch has been developed by Englyst et al. (5), in which samples are incubated with pancreatic α-amylase (PAA) and amyloglucosidase (AMG) for 2 h at 37°C. However, since literature reports indicate that the average time of residence of food in the human small intestine is ∼4 h (6, 7), this is the time of incubation that was employed in the current method. The levels of PAA and AMG used in the assay, and the incubation conditions employed, give resistant starch values for several control samples that are in line with values reported from ileostomy studies (8, 9). Starch that is digested during these incubation conditions represents a part of the total available carbohydrates that are absorbed in the human small intestine. In the current method, sucrose is specifically hydrolyzed to glucose and fructose by a sucrase enzyme that has no action on FOS (4, 10), and lactose is hydrolyzed to glucose and galactose by a specific β-galactosidase (MZ104, Megazyme, Bray, Ireland), which has limited action on galacto-oligosaccharides (GOS) of DP 3 and greater (11). Isomalto-oligosaccharides (IMO) are hydrolyzed by PAA/AMG to mostly glucose and isomaltose; isomaltose is an oligosaccharide that is digested in the human small intestine (4, 12, 13). In the current method, isomaltose is hydrolyzed to glucose using oligo-1,6-α-glucosidase. Galactose, glucose, and fructose are measured employing high purity and specific enzymes.

A single-laboratory validation was previously reported (4) with analysis performed on 55 different commercial food and beverage products, animal feeds, fruits and vegetables, and native and modified starches. Parameters examined during the validation included working range and linear range, selectivity, limit of detection (LOD), limit of quantification (LOQ), trueness (bias), precision (repeatability and intermediate precision), robustness, and stability. The method was accepted for First Action status in 2020 (*Official Method*^SM^**2020.07**), and a multi-laboratory collaborative study involving 17 laboratories and 10 matrixes was conducted and is discussed herein.

## Multi-laboratory Validation Study

### Practice Materials

Prior to the collaborative study, all participating laboratories were provided practice samples to familiarize themselves with the method and to ensure adequate method performance. Laboratories were shipped seven matrix samples along with *Official Method* **2020.07** required enzymes, a control sample, data reporting sheets, and an Excel calculator. Each laboratory was asked to perform a single analysis of each sample, to ask questions regarding the method, and to provide feedback to the Method Author. The samples included quick oats, infant formula powder (Cow and Gate, dairy based), red-skinned, yellow-fleshed potatoes, powdered cappuccino drink mix, granular RS_4_ resistant wheat starch, IMO mixture from non-genetically modified corn starch, and rye-based crispbread crackers.

- *Quick oats and rye crackers.—*Products were purchased from a local supermarket. Approximately 300 g of material was ground in a grinding mill until 100% passed through a 0.5 mm sieve. The ground material was collected in a plastic bag, mixed thoroughly by inversion, and then transferred to 1 L Duran bottles, well-sealed and stored at room temperature away from direct sunlight.
- *Potatoes.—*Potatoes were purchased from a local supermarket. They were steamed until tender, drained, and cooled to room temperature, chopped finely, weighed, and lyophilized, with wet and dry weights recorded. The recovered dry weight of potatoes was 18.1%.
- *Infant formula and cappuccino drink mix.—*Products were purchased from a local supermarket and transferred to well-sealed Duran glass bottles and stored at room temperature away from direct sunlight. These samples were analyzed as purchased.
- *Modified wheat starch and IMO.—*Modified wheat starch (phosphate cross-linked; RS_4_) was purchased from MGP Ingredients (Atchison, KS, USA), and IMO mixture was purchased from Top Health Ingredients (Edmonton, Alberta, Canada). These samples were analyzed as purchased.
  Test portions of ∼5 g of each sample type were transferred to prelabeled glass vials, which were sealed with rubber grommets and screw caps. Upon receipt, the collaborators were instructed to store all test portions at room temperature away from direct sunlight until use.
- *Moisture content of practice samples.—*Moisture contents were determined using an Ohaus MB45 Moisture analyzer. Values obtained were: quick oats 9.4%, infant formula 4.4%, dried potato 4.2%, cappuccino drink mix 2.3%, rye crackers 5.6%, phosphate cross-linked wheat starch 11.6%, and IMO 7.1%.

### Collaborative Study Materials

Ten pre-prepared foods were selected for the collaborative study to cover a broad range of food categories. These included bran cereal, chocolate digestive biscuits, small-dog adult kibble, chicken feed, ripe banana, cauliflower, mushrooms (closed cap), infant formula powder, milk-based hot chocolate drink mix, and instant pudding mix. Test portions of ∼5 g of each sample type were transferred to prelabeled glass vials, which were sealed with rubber grommets and screw caps. Two randomly selected test portion vials from each matrix preparation were packaged for shipment. Samples, a control bran cereal sample, copy of the method, a link to a video of the method, Excel-based data report forms, Mega-Calc™ Data Calculator v.12/20 (Megazyme, Bray, Ireland), sample storage instructions, and an adequate supply of enzymes in the K-AVCHO test kit as well as details on how to prepare and store them were distributed to collaborating laboratories by express overnight shipment. Upon receipt, the collaborators were instructed to store all test portions at room temperature away from direct sunlight until the start of the study and to store kit components as described on the individual bottle labels.

- *Bran cereal.—*The product was purchased from a local supermarket. Approximately 300 g of material was ground in a grinding mill until 100% passed through a 0.5 mm sieve. The ground material was collected in a plastic bag, mixed thoroughly by inversion, and then transferred to 1 L Duran bottles, well-sealed and stored at room temperature away from direct sunlight.
- *Chocolate digestive biscuits, small-dog adult kibble, and chicken feed.—*Products were purchased from a local supermarket and homogenized with a high-speed blender (e.g., Nutri-Bullet^**^®^**^). Portions of approximately 100 g were transferred to 2 L beakers, and approximately 800 mL of petroleum ether (or hexane) was added to each and stirred intermittently with a spatula in a well-ventilated fume cupboard over 15 min. The solids were allowed to settle over ∼4 h, and the supernatant solution was carefully decanted and discarded. This process was repeated a further two times. The solids were transferred to a flat polypropylene tray and allowed to dry in a well-ventilated fume hood over ∼3 h and then weighed. The content of fat remaining in the samples was determined using the ANKOM XT15 extractor. The dry material was ground until 100% passed a 0.5 mm sieve and then thoroughly mixed in a plastic bag and transferred and stored in well-sealed Duran glass bottles at room temperature away from direct sunlight. Chocolate biscuits, which had a fat content of 26.7%, were defatted as described above. Subsequent analysis of the dried product using the ANKOM XT15 extractor gave a residual fat content of 0.85%. Small-dog adult kibble feed, with a fat content of 8.0%, was extracted as described above, and subsequent analysis of the dried product using the ANKOM defatting equipment gave a residual fat content of 2.6%. Chicken feed, with a fat content of 10%, was extracted as described above. Subsequent analysis of the dried product using the ANKOM defatting equipment gave a residual fat content of 3.9%. These partially defatted samples were directly analyzed in the available carbohydrates assay procedure.
- *Banana, cauliflower, and mushrooms.—*Fresh produce was procured from a local supermarket. Cauliflower was steamed until tender, drained, and cooled to room temperature. Banana, steamed cauliflower, and mushrooms were chopped finely, weighed, and lyophilized, with wet and dry weights recorded. The recovered dry weight of mushrooms was 8.0%, steamed cauliflower was 11.1%, and ripe banana was 25.7%. Samples (∼300 g) were ground in a Nutri-Bullet homogenizer followed by further grinding in a mill until 100% passed through a 0.5 mm sieve. The ground material was thoroughly mixed in a plastic bag and transferred and stored in well-sealed Duran glass bottles at room temperature away from direct sunlight.
- *Infant formula powder, chocolate drink powder, and instant pudding powder.—*Samples were purchased from a local supermarket and transferred to well-sealed Duran glass bottles and stored at room temperature away from direct sunlight. These samples were analyzed as purchased.
- *Moisture content of collaborative study samples.—*Moisture contents were determined using an Ohaus MB45 Moisture analyzer. Values obtained were: bran cereal 4.3%, dried mushrooms 5.7%, defatted chocolate digestive biscuits 5.0%, defatted small-dog adult kibble 7.3%, infant formula 3.0%, dried cauliflower 5.4%, hot chocolate drink powder 3.0%, instant pudding mix 3.9%, dried ripe bananas 3.0%, and defatted chicken pellet feed 8.8%.

### Statistical Treatment

Results were submitted by collaborators using supplied Excel-based spreadsheets and evaluated according to AOAC guidelines (14) using an AOAC statistical workbook (15). Outlier results identified by the Cochran’s test for extremes of repeatability and the Grubb’s test for extremes of reproducibility were omitted from further calculations. Also determined were repeatability (s_r_) and reproducibility (s_R_) standard deviations, relative standard deviations of repeatability (RSD_r_) and reproducibility (RSD_R_), and measurement uncertainty (*U*) values.

**AOAC *Official Method*^SM^ 2020. 07**

**Determination of Available Carbohydrates in Cereal and Cereal Products, Dairy Products, Vegetables, Fruit, and Food Products** **and Animal Feeds**

**First Action 2020**

**Final Action 2022**

[Applicable for the determination of 0.18 to 100% (w/w) available carbohydrates, including digestible starch, maltodextrins, IMO, isomaltose, sucrose, lactose, glucose, fructose, and galactose, in cereal grains, breakfast cereals, bread, biscuits, pasta, dairy products, commercial starches and other polysaccharides, fruit (fresh and dried), vegetables (fresh and canned), and animal feeds (wet and dry)].

*See* Table **2020.07** for the results of the interlaboratory study supporting acceptance of this method.

**Table 2020.07.** Statistical evaluation interlaboratory study results for available carbohydrates (AVCHO method) with statistical outliers removed

	Bran cereal	Mushroom (closed cap)	Chocolate digestive biscuits	Small-dog adult kibble	Infant formula	Cauliflower	Hot chocolate drink mix	Instant pudding mix	Ripe banana	Chicken feed
Samples	A & E	D & N	B & K	F & O	C & S	I & P	G & H	J & R	L & Q	M & T
No. of laboratories	17	17	17	17	16	17	17	17	17	16
No. of replicates	33	34	33	34	32	34	33	34	34	31
Mean, g/100 g	46.01	2.15	81.89	47.28	58.01	27.21	74.89	71.92	55.09	42.20
S_r_, g/100 g	0.62	0.08	1.51	1.29	1.81	0.94	1.53	2.23	1.28	0.52
S_R_, g/100 g	1.67	0.25	2.94	2.31	2.04	1.68	4.24	2.50	2.58	1.81
RSD_r_, %	1.35	3.91	1.84	2.73	3.12	3.46	2.05	3.10	2.32	1.23
RSD_R_, %	3.63	11.58	3.59	4.88	3.51	6.19	5.67	3.47	4.68	4.28

## A. Principle

Test samples to be analyzed are suspended in maleate buffer (pH 6.0) containing PAA and AMG and continually mixed or stirred at 37°C for 4 h. During this time, digestible starch is hydrolyzed to glucose plus traces of remaining maltose as shown in Equation 1.


(1) $$\text{Digestible starch}\overset{\left(\text{PAA}+\text{AMG},\;\text{pH}\;6,\;37{}^{\circ}C,\;4\;h\right)}{\to }\text{glucose}\;\left(\text{plus traces of maltose}\right)$$

Samples of the solutions are then removed, diluted, and centrifuged. Aliquots of this solution are incubated with a mixture of sucrase, maltase, β-galactosidase, and oligo-1,6-α-glucosidase at pH 6.5 and 30°C for 30 min, during which time sucrose is specifically hydrolyzed to glucose and fructose by sucrase enzyme, maltose and isomaltose are hydrolyzed to glucose, and lactose is hydrolyzed to galactose and glucose by β-galactosidase (Equations 2, 3, 4, and 5).


(2) $$\text{Sucrose}\overset{\left(\text{sucrase},\;\text{pH}\;6.5,\;30{}^{\circ}C,\;30\;\text{min}\right)}{\to }\text{glucose}+\text{fructose}$$

(3) $$\text{Maltose}\overset{\left(\text{maltase},\;\text{pH}\;6.5,\;30{}^{\circ}C,\;30\;\text{min}\right)}{\to }\text{glucose}$$

(4) $$\text{Isomaltose}\overset{\left(\text{oligo}-1,6-\alpha -\text{glucosidase},\;\text{pH}\;6.5,\;30{}^{\circ}C,\;30\;\text{min}\right)}{\to }\text{glucose}$$

(5) $$\text{Lactose}\overset{\left(\beta -\text{galactosidase},\;\text{pH}\;6.5,\;30{}^{\circ}C,\;30\;\text{min}\right)}{\to }\text{galactose}+\text{glucose}$$

In the presence of the enzymes galactose dehydrogenase (GalDH) and galactose mutarotase (GalM), β-D-galactose is oxidized by nicotinamide-adenine dinucleotide phosphate (NADP^+^) to D-galactonate with the formation of reduced nicotinamide-adenine dinucleotide phosphate (NADPH) (Equation 6).


(6) $$\beta -D-\mathrm{Galactose}+\mathrm{NAD}P^{+}\overset{\left(\mathrm{GalDH}/\mathrm{GalM},\;pH\;7.6,\;30{}^{\circ}C,\;3\;\min\right)}{\to }g\mathrm{alactonate}+\mathrm{NADPH}+H^{+}$$

The amount of NADPH formed in this reaction is stoichiometric with the amount of D-galactose. It is the NADPH that is measured by the increase in absorbance at 340 nm.

Glucose and fructose are phosphorylated by the enzyme hexokinase (HK) and adenosine-5′-triphosphate (ATP) to glucose-6-phosphate (G-6-P) and fructose-6-phosphate (F-6-P) with the simultaneous formation of adenosine-5′-diphosphate (ADP) (Equations 7 and 8).


(7) $$\mathrm{Glucose}+\mathrm{ATP}\overset{\left(HK,\;pH\;7.6,\;30{}^{\circ}C,\;5\;\min\right)}{\to }G-6-P+\mathrm{ADP}$$

(8) $$\mathrm{Fructose}+\mathrm{ATP}\overset{\left(HK,\;pH\;7.6,\;30{}^{\circ}C,\;5\;\min\right)}{\to }F-6-P+\mathrm{ADP}$$

In the presence of the enzyme glucose-6-phosphate dehydrogenase (G6P-DH), G-6-P is oxidized by nicotinamide-adenine dinucleotide phosphate (NADP^+^) to gluconate-6-phosphate with the formation of reduced nicotinamide-adenine dinucleotide phosphate (NADPH) (Equation 9).


(9) $$G-6-P+\mathrm{NAD}P^{+}\overset{\left(G6P-DH,\;pH\;7.6,\;30{}^{\circ}C,\;5\;\min\right)}{\to }g\mathrm{luconate}-6-\mathrm{phosphate}+\mathrm{NADPH}+H^{+}$$

The amount of NADPH formed in this reaction is stoichiometric with the amount of glucose. It is the NADPH that is measured by the increase in absorbance at 340 nm.

On completion of Equation 8, F-6-P is converted to G-6-P by phosphoglucose isomerase (PGI) (Equation 10).


(10) $$F-6-P\overset{\left(\mathrm{PGI},\;pH\;7.6,\;30{}^{\circ}C,\;10\;\min\right)}{\to }G-6-P$$

The G-6-P formed reacts in turn with NADP^+^, forming gluconate-6-phosphate and NADPH, leading to a further rise in absorbance that is stoichiometric with the amount of fructose.

The method is simple to use, and the absorbance response for galactose, glucose, and fructose is the same (Figure **2020.07A**).

**Figure 2020.07A.**
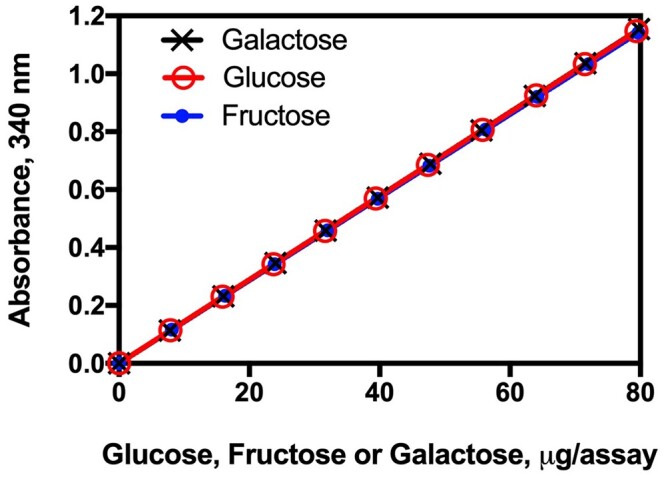
Linearity of the absorbance response in the enzymatic reactions in the measurement of galactose, glucose, and fructose. r^2^ = 0.9999 for each sugar.

## B. Chemicals and Reagents

- *EtOH.—*95% v/v.
- *Imidazole buffer (2 M, pH 7.6) containing magnesium chloride (100 mM) and sodium azide (0.02% w/v) as a preservative.—*Stable for >2 years at 4°C. Use as supplied.
- *NADP^+^ (0.84 mM) plus ATP (2.5 mM).—*Stable for >5 years stored dry below −10°C. Dissolve the contents of the vial in 12 mL of distilled water and store in ∼4 mL aliquots at <−10°C between use.
- *Stock PAA plus AMG powder.—*PAA (40 KU/g) plus AMG (17 KU/g) as a freeze-dried powder mixture. *Note*: One unit of AMG activity is the amount of enzyme required to release one µmole of d-glucose from soluble starch per minute at 40°C and pH 4.5; one unit of PAA activity is the amount of enzyme required to release one µmole of *p*-nitrophenyl from Ceralpha reagent per minute at 40°C and pH 6.9 (*Official Method* **2002.01**). PAA/AMG preparations should be essentially devoid of β-glucanase, β-xylanase, and detectable levels of free d-glucose. Stable for >4 years at −20°C.
- *PAA/AMG solution.—(1)* Immediately before use, dissolve 0.5 g of PAA/AMG powder **[B(d)]** in 25 mL of sodium maleate buffer (50 mM, pH 6.0) **[B(l)]** and stir for approximately 5 min. This preparation **[B(e)***(1)***]** contains PAA at 0.8 KU/mL and AMG at 0.34 KU/mL. Store on ice during use. Use on the day of preparation. *(2)* Alternatively: Some individuals are allergic to powdered PAA and/or AMG. In this instance, engage an analyst who is not allergic to prepare the powdered enzymes as an ammonium sulfate suspension as follows: gradually add 2.5 g of PAA/AMG powder mix (PAA 40 KU/g plus AMG 17 KU/g) to 35 mL sodium maleate buffer (50 mM, pH 6.0) **[C(l)]** in a 100 mL beaker on a magnetic stirrer in a laboratory hood and stir until the enzymes are completely dissolved (∼5 min). Add 17 g of granular ammonium sulfate and dissolve by stirring. Adjust the volume to 50 mL with ammonium sulfate solution **[B(n)]** and store at 4°C. This preparation **[B(e)***(2)***]** contains PAA at 2 KU/mL and AMG at 0.85 KU/mL. Stable at 4°C for 3 months.
- *Sucrase (85 U/mL on sucrose), maltase (500 U/mL on p-nitrophenyl α-glucoside), β-galactosidase (500 U/mL on p-nitrophenyl β-D-galactoside) plus oligo-a-1,6-glucosidase (500 U/mL on on p-nitrophenyl α-glucoside).—*Suspension in 3.2 M ammonium sulfate plus sodium azide (0.02%, w/v). Stable for >2 years at 4°C. Use as supplied.
- *Galactose dehydrogenase (200 U/mL) plus galactose mutarotase suspension (4.1 mg/mL*)*.—*Suspension in 3.2 M ammonium sulfate plus sodium azide (0.02%, w/v). Stable for >2 years at 4°C. Use as supplied.
- *Hexokinase (425 U/mL) plus glucose-6-phosphate dehydrogenase (110 U/mL) .—*Suspension in 3.2 M ammonium sulfate plus sodium azide (0.02%, w/v). Stable for >2 years at 4°C. Use as supplied.
- *Phosphoglucose isomerase suspension (1000 U/mL).—*Suspension in 3.2 M ammonium sulfate plus sodium azide (0.02%, w/v). Stable for >2 years at 4°C. Use as supplied.
- *D-Glucose, D-fructose plus D-galactose standard solution (5 mL, 0.2 mg/mL of each sugar in 0.02%, w/v sodium azide solution*)*.—*Stable for >2 years at 4°C. Use as supplied.
- *Available carbohydrates control (∼10 g).—*Available carbohydrates value shown on the label. Stable for >2 years at 4°C.
- *Sodium maleate buffer (50 mM, pH 6.0) plus CaCl_2_ (2 mM).*—Dissolve 11.6 g of maleic acid in 1600 mL of deionized water and adjust the pH to 6.0 with 4 M (160 g/L) NaOH solution. Add 0.6 g of calcium chloride dihydrate (CaCl_2_·2H_2_O), dissolve, and adjust the volume to 2 L. Store in a well-sealed bottle (e.g., Duran^®^), and add two drops of toluene to prevent microbial infection. Stable for ∼1 year at 4°C.
- *Sodium maleate buffer (50 mM, pH 6.5) containing BSA (0.5 mg/mL).*—Dissolve 5.8 g of maleic acid in 800 mL of deionized water and adjust the pH to 6.5 with 4 M (160 g/L) NaOH solution. Add 0.5 g of BSA (bovine serum albumin; Sigma Chemical Co. Cat. No. A2153-100G) and 0.2 g of sodium azide and dissolve by stirring. Adjust the volume to 1 L. Store in a well-sealed bottle (e.g., Duran). Stable for ∼1 year at 4°C.
- *Ammonium sulfate solution, 50% w/v.*—Add 50 g of ammonium sulfate to 80 mL of distilled water and dissolve by stirring. Adjust volume to 100 mL with distilled water. Store in a well-sealed bottle (e.g., Duran). Stable for >2 years at room temperature.

Items **B**(**b**) to **B**(**k**) are supplied in the Available Carbohydrates Assay Kit (K-AVCHO) available from Megazyme, Bray Business Park, Southern Cross Road, Bray, County Wicklow, Ireland.

## C. Apparatus

- *Grinding mill.—*Centrifugal, with 12-tooth rotor and 0.5 mm sieve. Alternatively, a cyclone mill can be used for small test laboratory samples provided they have sufficient air flow or other cooling to avoid overheating samples.
- *Meat mincer.*—Hand-operated or electric, fitted with a 4.5 mm screen.
- *Water bath*.—Linear motion, shake speed 200 strokes/min, 35 mm stroke length, capable of maintaining temperature of 37 ± 1°C. With clips or springs to allow attachment of tubes. http://www.keison.co.uk/grantinstruments_ols200.shtml (Figure **2020.07B**). Shaking speed must be sufficient to keep the sample completely suspended during the incubation period. This is the preferred method for sample incubation.
- *Water bath.*—To accommodate a submersible magnetic stirrer with an immersion heater (e.g., Julabo^®^ Immersion Circulator, Julabo, Seelbach, Germany) (Figure **2020.07C**). This is an alternative method for sample incubation.
- *Submersible magnetic stirrer.*—2mag Mixdrive 15^®^ submersible magnetic stirrer (Munich, Germany) or equivalent set at 170 rpm.
- *Spectrophotometer.*—Capable of operating at 340 nm, 10 mm path length, and maintaining 30 ± 1°C; e.g., MegaQuant^TM^ Wave Spectrophotometer (Megazyme cat. no. D-MQWAVE).
- *Analytical balance.*—0.1 mg readability, accuracy, and precision.
- *Freeze-drier.*—Virtis Genesis^®^ 25XL Biopharma Process Systems, Biopharma House, Winchester, UK, or similar.
- *Microfuge centrifuge.*—Capable of 13 000 rpm (∼15 500 × *g*).
- *Disposable microfuge tubes*.—2.0 mL polypropylene with attached snap cap.
- *pH meter.*
- *Vortex mixer.*
- *Moisture analyzer.*—OHAUS MB45, or equivalent.
- *Fat extraction system.*—ANKOM XT15 Extractor, or equivalent.
- *Magnetic stirrer.*
- *Magnetic stirring bars.*—Polytetrafluoroethylene, 20 × 6 mm, ridged.
- *Digestion bottles.*—250 mL soda-lime glass, wide-mouth bottles with polyvinyl lined cap (e.g., Fisherbrand^®^ cat. no. FB73219).
- *Laboratory timer.*
- *Micro-pipettors.*—Capable of dispensing 1.00 mL, 100 µL, and 20 µL for dispensing 1.0 mL and 100 µL of sample solutions and 20 µL of enzyme preparations.
- *Positive displacement pipettors.*—With a 5 mL tip to dispense 0.5 mL of 95% v/v and to dispense 0.1 mL sucrase/β-galactosidase, 0.1 mL imidazole buffer, and 0.1 mL NADP^+^ solution; with a 25 mL tip to dispense 2.5 mL aliquots of PAA/AMG; and with a 12.5 mL tip to remove 1.0 mL aliquots from incubation solutions.
- *Dispensers.*—Brand HandyStep dispensette^®^ S Digital 2.5–25 mL Cat. No. 4600351, or equivalent, to dispense 17.5 mL of sodium maleate buffer [**B(l)**] and 25 mL of distilled water.
- *Polypropylene tubes.*—30 mL, 30 × 84 mm, flat-bottom centrifuge tubes with screw cap.
- *Polypropylene sheet with precision cut holes.*—To hold and align 30 mL polypropylene tubes on the stirrer plate of the 2mag Mixdrive 15 submersible magnetic stirrer (Figure **2020.07C**).

**Figure 2020.07B.**
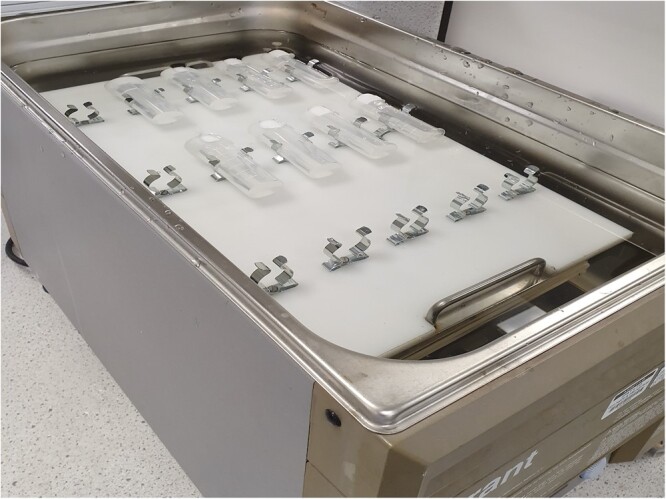
Polypropylene tube-holder in a Grant OLS 200 water bath to attach 30 mL polypropylene tubes containing samples.

**Figure 2020.07C.**
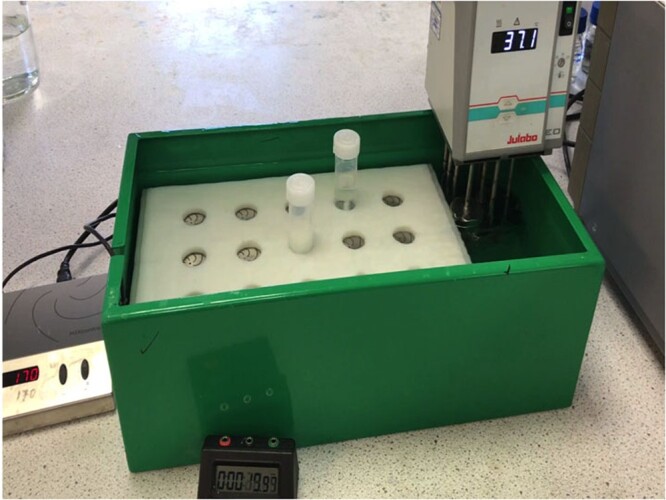
Designed water bath (Cat. No. D-TDFBTH) with submersible 2mag Mixdrive 15 submersible magnetic stirrer and a designed polypropylene tube holder (Megazyme Cat. No. D-PPTH) [C(w)]. Samples (∼0.5 g) incubated with enzyme mixture in 30 mL polypropylene tubes with stirrer bar.

## D. Safety Considerations

The general safety measures that apply to all chemical substances should be adhered to. For more information regarding the safe use and handling of the Available Carbohydrates Assay Kit reagents (K-AVCHO), refer to the K-AVCHO SDS document that is downloadable from the Megazyme website (https://www.megazyme.com/available-carbohydrates-assay-kit?c=110). Some individuals are allergic to powdered pancreatic α-amylase and/or amyloglucosidase. This enzyme preparation should be weighed and dissolved in a well-ventilated fume hood. The preparation can be stabilized with ammonium sulfate to reduce handling of the powder product.

## E. Preparation of Test Samples

Collect and prepare food materials as “intended to be eaten,” i.e., cook pasta and potatoes. For dry foods, animal feeds, and breakfast cereals, grind an approximately 50 g sample in a grinding mill to pass a 0.5 mm sieve. Transfer all materials into wide-mouthed plastic jars, seal, and mix well by shaking and inversion. Freeze-dry high-moisture (>25%, w/w) materials. Pour canned vegetables onto a strainer and wash with demineralized water, freeze-dry, and mill to pass a 0.5 mm screen. Homogenize dried fruit materials in a high-speed blender (e.g., Nutri-Bullet) and then dry the paste in a forced-air oven at 40°C overnight. Further grind samples to be analyzed using a mortar and pestle. Homogenize high-fat samples (>5% fat content) such as malted-milk biscuits, chocolate digestive biscuits, jam and cream biscuits, shortbread finger biscuits, dairy milk chocolate, and pet foods using a high-speed blender (e.g., Nutri-Bullet). In a well-ventilated fume hood, transfer a sample of the homogenized material (∼10 g, weighed accurately) to a preweighed 200 mL beaker and add 50 mL of petroleum ether. Stir the mixture with a spatula for 20 s, and allow the solids to settle. Carefully decant the supernatant solution and then repeat this process a further two times. Allow the solids in the beaker to dry in a well-ventilated fume hood and weigh. Calculate fat content. Store materials in the presence of a desiccant.

## F. Measurement of Enzyme Activities (Enzymes Are Supplied Ready to Use in the Megazyme Available Carbohydrates Assay Kit; There Is No Requirement to Perform the Operations Shown Below)

Measure the activity of α-amylase in PAA using the Ceralpha^**^®^**^ assay procedure employing nonreducing end-blocked *p*-nitrophenyl maltohepatoside in the presence of excess levels of thermostable α-glucosidase. Perform incubations in sodium maleate buffer at pH 6.9 and 40°C as described in the α-Amylase Assay Kit (Ceralpha Method) booklet (Megazyme Cat. No. K-CERA; AOAC *Official Method* **2002.01**). One unit of enzyme activity is defined as the amount of enzyme that releases one µmole of *p*-nitrophenol per minute under the defined assay procedure. Report the α-amylase activity at the optimal pH of 6.9. Incubations for the measurement of digestible starch, resistant starch, and available carbohydrates are performed at pH 6.0. α-Amylase activity at pH 6.0 is ∼ 77% of that at pH 6.9. Assay AMG by incubating 0.2 mL of suitably diluted enzyme in 100 mM sodium acetate buffer (pH 4.5) with 0.5 mL of soluble starch (10 mg/mL) in 100 mM sodium acetate buffer (pH 4.5) at 40°C. At various time intervals, heat reaction tubes to ∼100°C in a boiling water bath to terminate the reaction, and measure released glucose using GOPOD reagent (Glucose Assay Kit; GOPOD Format; Megazyme Cat. No. K-GLUC). One unit of AMG is defined as the amount of enzyme required to release one µmole of D-glucose per minute at pH 4.5 and 40°C. When in admixture with PAA, assay AMG using AMG Assay Reagent (Megazyme Cat. No. R-AMGR3) and calculate units of activity on starch using a conversion factor. Record the AMG activity at the optimal pH of 4.5. Incubations for the measurement of digestible starch, resistant starch, and available carbohydrates are performed at pH 6.0. The AMG activity at pH 6.0 is ∼60% of that at pH 4.5.

## G. Enzyme Digestion of Sample

- *Hydrolysis of digestible starch.—(1)* Weigh a 0.500 ± 0.005 g test portion accurately into a 30 mL tube **[C(v)]**. *(2)* Wet the test portion with 0.5 mL of ethanol (95%, v/v) **[C(a)]**, add 17.5 mL of 50 mM sodium maleate buffer, pH 6.0 **[B(l)],** using a positive displacement dispenser **[C(u)],** and vortex mix the tube for a few minutes to ensure all sample is suspended, and incubate the tubes at 37°C for 5 min. *(3)* If incubations are to be performed in a shaking water bath, add 2.5 mL of PAA/AMG solution **[C(e)***(1)***]** to the tube, cap the tube, and place the tubes horizontally in the line of direction of shaking **[C(c)]** (Figure **2020.07B**), set at 200 strokes per min, and incubate at 37°C for 4 h. *(4)* If incubations are to be performed by stirring of the tube contents, add a magnetic stir bar **[C(p)]** and 2.5 mL of PAA/AMG solution **[B(e)***(1)***]** to the tube, cap the tube, and incubate the reaction solution with stirring at 170 rpm on a submersible magnetic stirrer **[C(e)]** at 37°C for 4 h (Figure **2020.07C**) using a precision-cut polypropylene sheet **[C(w)]** to hold the tubes in place.
*Note*: If using an (NH_4_)_2_SO_4_ suspension of this enzyme preparation **[B(e)***(2)***]**, add 19 mL of 50 mM sodium maleate buffer **[B(l)]** to the test portion, mix the contents on a vortex mixer, and incubate the tubes at 37°C for 5 min. Then add 1 mL of enzyme suspension. *(5)* After 4 h, remove the tubes from the shaking or stirring water bath and filter the reaction solution through glass fiber filter paper. Alternatively, store the reaction solutions at room temperature for 5 min to allow solids to partially settle and then transfer 2 mL of the supernatant solution **[G(a)***(5)***]** to a 2.0 mL polypropylene microfuge tube **[C(j)]** and centrifuge at 13 000 rpm (15 500 × *g*) for 5 min. Accurately transfer 1.0 mL of the filtered solution or supernatant on centrifugation to 25 mL of distilled water in a 30 mL polypropylene tube **[C(v)]**. Cap the tube, mix the contents thoroughly, and store at 4°C awaiting analysis. For materials containing <10% available carbohydrate content, carefully transfer 1.0 mL of the reaction solution to 5 mL of distilled water and mix well.
- *Measurement of available carbohydrates.*—Analyze a 0.1 mL aliquot of the supernatant solution as described in Figure **2020.07D**. Concurrently, analyze 0.1 mL of the D-glucose/D-fructose/D-galactose standard solution (Bottle 6 in the K-AVCHO kit). In this assay, lactose is hydrolyzed to glucose plus galactose by α-galactosidase, sucrose is hydrolyzed to glucose and fructose by the sucrase enzyme (which has no action on fructo-oligosaccharides; FOS), remaining traces of maltose are hydrolyzed to glucose by maltase, and isomaltose is hydrolyzed to glucose by oligo-α-1,6-glucosidase. Determine free galactose, glucose, and fructose by the successive increase in absorbance as shown in Figure **2020.07E**.

**Figure 2020.07D.**
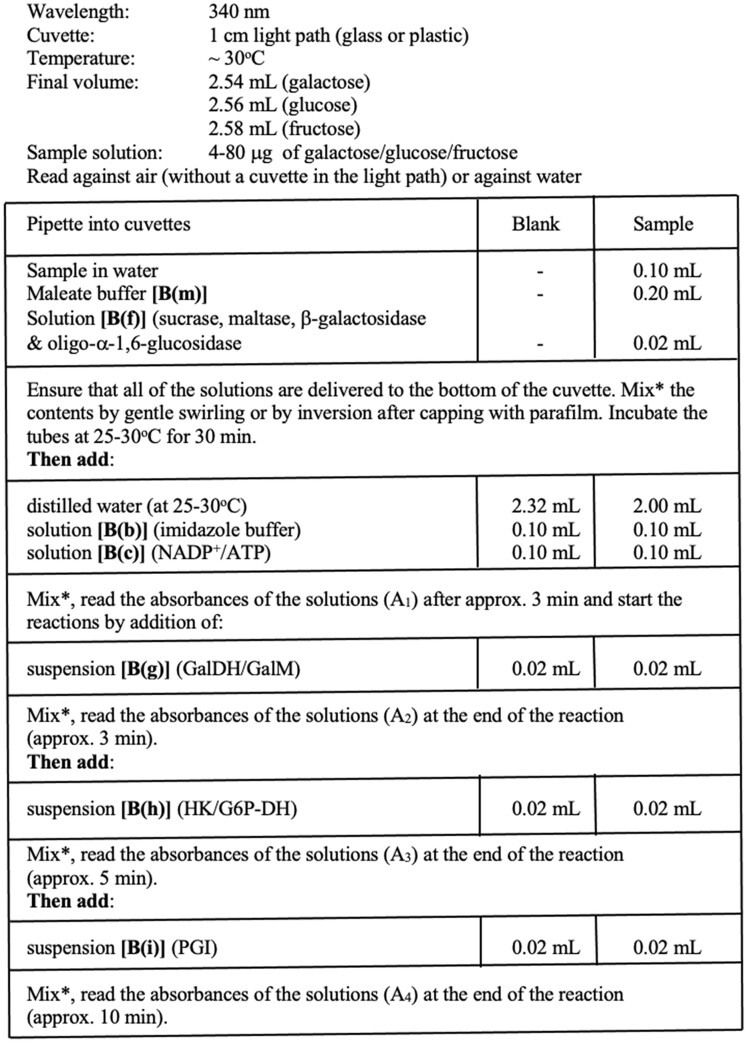
Procedure for the sequential measurement of galactose, glucose, and fructose in a spectrophotometer cuvette.

**Figure 2020.07E.**
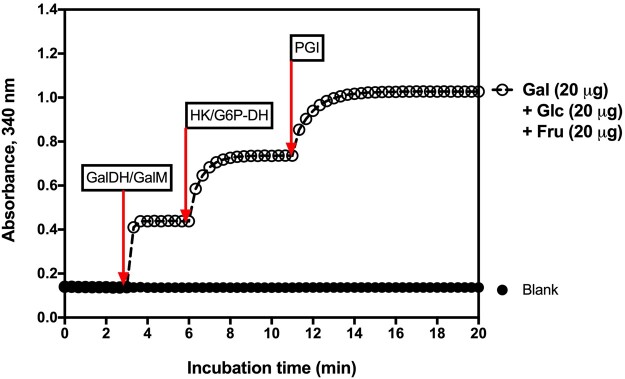
Increase in absorbance on incubation of a mixture of galactose, glucose, and fructose with specific enzymes in the presence of NADP^+^ and ATP, as detailed in Figure **2020.07D**.

## H. Determination of Galactose, Glucose, Fructose, and Available Carbohydrates

- Determine the absorbance difference (A_2_-A_1_) for both blank and sample. Subtract the absorbance difference of the blank from the absorbance difference of the sample, thereby obtaining ΔA_galactose_.
- Determine the absorbance difference (A_3_-A_2_) for both blank and sample. Subtract the absorbance difference of the blank from the absorbance difference of the sample, thereby obtaining ΔA_glucose_.
- Determine the absorbance difference (A_4_-A_3_) for both blank and sample. Subtract the absorbance difference of the blank from the absorbance difference of sample, thereby obtaining ΔA_fructose_.
- The values of ΔA_galactose_, ΔA_glucose_, and ΔA_fructose_ should as a rule be at least 0.100 absorbance units to achieve sufficiently accurate results. If values are less than this, decrease the dilution of the sample extract or increase the volume of the aliquot analyzed and reassay. This may not be possible if the combined absorbance value then falls outside the analytical range of the assay. If the combined absorbance value is above 1.40, dilute the sample further and reassay. If the combined absorbance value is less than 0.40, increase the volume of sample analyzed from 0.1 mL to 0.5 mL and decrease the volume of water added to the cuvette from 2.0 mL to 1.6 mL.
- Calculate the concentration of galactose, glucose, and fructose as follows:

$$c=\frac{V\;\times \;MW}{\varepsilon \;\times \;d\;x\;v}\times \;\Delta A\;\times \;D\left[g/L\right]$$

where: V = final volume [mL]; MW = molecular weight of D-galactose, D-glucose, or D-fructose [g/mol]; ε = extinction coefficient of NADPH at 340 nm = 6300 [l × mol^−1^ × cm^−1^]; d = light path [cm]; v = sample volume [mL]; D = dilution factor (26-fold) (or 6-fold for samples with <10% available carbohydrates)

It follows for galactose:


$$\begin{array}{cc} c=\frac{2.54\;\times \;180.16}{6300\;\times \;1.0\;\times \;0.1}\times \;\Delta A_{\mathrm{galactose}}\;\times \;26 & \left[g/L\right]\\ =1\mathrm{8.885}\;\times \;\Delta A_{\mathrm{galactose}} & \left[g/L\right] \end{array}$$

for glucose:


$$\begin{array}{cc} c=\frac{2.56\;\times \;180.16}{6300\;\times \;1.0\;\times \;0.1}\times \;\Delta A_{\mathrm{glucose}}\;\times \;26 & \left[g/L\right]\\ =1\mathrm{9.034}\;\times \;\Delta A_{\mathrm{glucose}} & \end{array}$$

for fructose:


$$\begin{array}{cc} c=\frac{2.58\;\times \;180.16}{6300\;\times \;1.0\;\times \;0.1}\times \;\Delta A_{\mathrm{fructose}}\;\times \;26 & \left[g/L\right]\\ =1\mathrm{9.183}\;\times \;\Delta A_{\mathrm{fructose}} & \left[g/L\right] \end{array}$$

- Calculate the content (g/100 g) in solid and semi-solid materials as follows:

Content of galactose:


$$=c_{\mathrm{galactose}}\left[g/L\right]\;\times \frac{EV}{1000}\times \frac{1}{W}\times \;100\;\left[g/100\;g\right]$$

Content of glucose:


$$=c_{\mathrm{glucose}}\left[g/L\right]\;\times \frac{EV}{1000}\times \frac{1}{W}\times \;100\left[g/100\;g\right]$$

Content of fructose:


$$=c_{\mathrm{fructose}}\left[g/L\right]\;\times \frac{EV}{1000}\times \frac{1}{W}\times \;100\left[g/100\;g\right]$$

where: c_galactose_ [g/L] = concentration of galactose per L of undiluted extraction solution; c_glucose_ [g/L] = concentration of glucose per L of undiluted extraction solution; c_fructose_ [g/L] = concentration of fructose per L of undiluted extraction solution; EV = volume of solution used in the initial extraction (i.e., 20.5 mL); EV/1000 = adjustment from g/L of undiluted extraction solution to g/volume of extraction solution actually used; W = weight of test portion analyzed in g.

*Available carbohydrates* (g/100 g)

= *galactose* (g/100 g) + *glucose* (g/100 g) + *fructose* (g/100 g)

*Note*: These calculations can be simplified by using the Megazyme Mega-Calc^TM^ downloadable workbook from the Megazyme website (https://www.megazyme.com/available-carbohydrates-assay-kit?c=110).

## I. Indicative Controls

Indicative controls are used as a check on assay conditions. Available carbohydrates, galactose, glucose, and fructose values for the control sample included in the Available Carbohydrates Assay Kit should be within 3% of that stated on the label. Inulin, levan, and galactosyl-sucrose oligosaccharides should not be hydrolyzed and thus should contribute nothing to the available carbohydrates value. Sucrose in FOS will be hydrolyzed by the sucrase and, thus, correctly measured as available carbohydrates.

## Results and Discussion

Samples were selected to cover a wide range of foods, feeds, and ingredients for which available carbohydrate values would be useful. The samples chosen contained various levels of lactose, sucrose, starch, and nonresistant starch to ensure complete and reproducible hydrolysis of these components to the monosaccharides, galactose, glucose, and fructose and to demonstrate quantitative measurement of the three monosaccharides. All materials were prepared, dried, dispensed into sealed tubes, and blind-coded before dispatch to avoid possible deterioration during shipping.

### Practice Sample Results

The results of the analyses of the practice samples for total available carbohydrates are shown in Table 1. The results were typical for manual enzymatic methods. Reproducibility values were in the range of performance characteristics typically found for enzymatic methods, wherein a significant number of manual steps are necessary to perform the assay. The reproducibility standard deviation (s_R_) ranged from 1.37 to 3.19 g/100 g, and the reproducibility relative standard deviation (RSD_R_) ranged from 2.7 to 11.4%, with 7 of the 8 samples having an RSD_R_ of <4.4%. The higher reproducibility standard deviation for RS_4_ (phosphate crosslinked wheat starch) most likely reflects the different incubation/suspension conditions of the samples with pancreatic α-amylase plus amyloglucosidase employed by some of the collaborators. The reproducibility values are consistent with those reported for analyses of similar samples with dietary fiber methods.

**Table 1.** Practice sample data for the AVCHO method for available carbohydrates

Collaborator	Sample and determined available carbohydrates (g/100 g, as is)^a^							
	P1	P2	P3	P4	P5	P6	P7	Control sample
1	64.9	56.3	70.6	69.0	33.5	84.9	53.7	46.4
2	66.6	56.5	75.2	71.8	27.1	87.9	55.0	46.4
3	65.9	57.5	75.8	75.2	26.9	90.4	54.3	47.3
4	62.0	57.1	71.8	68.4	26.4	87.9	51.4	43.8
5	69.3	60.2	80.3	76.4	28.9	91.0	59.2	49.5
6	66.1	55.8	75.2	67.6	28.9	87.1	54.7	46.4
7	72.6	55.4	75.3	71.5	31.1	84.5	52.6	47.9
8	66.9	56.4	73.5	72.9	26.7	85.9	55.1	46.6
9	67.9	59.5	75.5	73.1	25.5	87.1	55.8	ND^b^
10	66.9	56.7	74.2	71.3	30.1	88.1	55.1	47.4
11	63.4	57.3	71.2	69.9	31.4	84.0	51.5	46.8
12	67.4	56.7	75.1	74.8	22.7	87.9	54.8	47.2
13	64.6	56.3	74.5	73.6	23.4	87.2	54.3	45.3
14	68.4	57.7	75.2	74.0	25.2	89.3	56.2	47.7
15	68.2	51.3	77.7	76.1	26.8	90.0	56.9	49.0
16	68.6	62.4	74.5	67.8	27.9	90.4	59.9	ND
17	64.3	56.3	71.5	71.1	33.9	83.2	51.5	47.7
Mean, g/100 g	66.7	57.0	74.5	72.0	28.0	87.5	54.8	47.0
S_R_, g/100 g	2.50	2.31	2.40	2.83	3.19	2.36	2.41	1.37
RSD_R_, %	3.7	4.0	3.2	3.9	11.4	2.7	4.4	2.9

a P1 = quick oats. P2 = infant formula. P3 = red potatoes. P4 = cappuccino drink mix. P5 = RS_4_ wheat starch. P6 = isomalto-oligosaccharides. P7 = rye crackers. Control sample is a bran cereal.

b ND = Not determined.

### Collaborative Study Results

All 17 laboratories that analyzed practice samples completed the study and reported a full set of results. Collaborating laboratory data were evaluated statistically according to AOAC International Guidelines (14) using an AOAC-supplied workbook (15). Of the 10 valid pairs of assay results reported from 17 laboratories for available carbohydrates, laboratories 1 through 14 had no statistical outliers, and laboratories 15, 16, and 17 had 8 statistical outliers, including 2 statistical outlier pairs (*see*Table 2). The raw data for available carbohydrates and individual sugars by laboratory are shown in Tables 2–5, and statistical analyses with and without outliers removed are shown in Tables 6 and **2020.07**. Outliers and their statistical basis are indicated and footnoted in Table 2.

**Table 2.** Interlaboratory study results for total available carbohydrates

Laboratory no.	Available carbohydrates, g/100 g																			
	Bran cereal		Mushroom (closed cap)		Chocolate digestive biscuits		Small dog adult kibble		Infant formula		Cauliflower		Hot chocolate drink mix		Instant pudding mix		Ripe banana		Chicken feed	
	A	E	D	N	B	K	F	O	C	S	I	P	G	H	J	R	L	Q	M	T
1	44.9	46.5	1.9	1.9	78.4	77.9	47.9	45.9	58.0	56.6	26.9	27.8	76.7	75.4	72.0	70.0	54.6	54.4	42.4	41.7
2	46.4	45.9	2.0	2.0	81.8	82.5	47.3	47.0	60.8	57.9	27.6	27.6	74.2	74.5	70.3	72.4	55.8	55.2	42.1	41.0
3	49.6	48.8	2.3	2.2	85.9	86.9	52.6	49.5	53.9	60.6	28.4	29.4	77.7	80.0	75.3	73.7	54.8	55.5	43.9	43.1
4	44.2	43.1	2.0	2.0	78.4	78.1	44.0	43.8	54.5	53.9	25.8	26.2	70.9	71.6	70.4	67.9	51.9	50.8	38.6	38.4
5	45.4	46.1	1.9	2.0	81.5	81.3	46.7	46.4	57.8	58.0	28.4	28.0	76.5	73.0	72.0	73.0	54.6	55.3	40.9	41.2
6	45.2	45.4	2.1	2.0	80.6	80.5	46.4	48.8	57.2	57.1	27.2	27.4	76.9	74.1	71.4	70.3	53.8	54.3	41.3	40.6
7	47.9	47.5	2.4	2.3	82.9	85.0	49.3	51.1	59.9	59.9	28.2	29.6	73.5	71.4	70.1	75.6	61.7	57.4	46.4	46.7
8	43.9	44.2	2.5	2.5	77.7	79.0	44.6	45.4	58.2	54.8	26.6	28.3	80.0	77.2	70.9	72.3	54.6	52.6	41.8	41.7
9	46.7	46.8	2.1	2.3	82.5	83.2	47.8	47.5	58.9	58.2	24.8	25.8	73.2	75.0	72.5	72.0	56.9	55.5	42.9	42.7
10	46.6	46.2	2.3	2.4	82.0	82.0	47.7	47.2	58.3	58.0	27.3	27.3	74.8	74.7	70.4	71.1	55.3	55.8	40.6	41.7
11	44.2	42.8	1.9	2.1	76.1	80.9	43.3	44.4	59.1	54.2	29.5	27.1	70.6	73.0	78.4	70.5	59.1	57.8	42.3	42.1
12	47.3	47.2	2.0	2.0	84.4	87.0	48.6	48.0	58.8	56.9	27.9	28.0	79.9	77.9	71.8	72.2	55.0	55.5	43.3	41.7
13	46.2	46.3	2.0	2.0	81.5	84.0	47.8	47.3	58.2	58.4	27.0	27.6	77.6	77.3	74.6	72.5	54.2	55.0	41.8	42.2
14	48.2	48.1	2.3	2.0	84.5	84.8	48.5	47.8	59.8	59.3	28.6	27.9	78.4	79.0	73.6	73.3	57.6	57.3	42.4	42.4
15	44.9	43.3	2.2	2.3	80.2	70.2^a^	46.5	42.6	52.3^a^	67.5^a^	23.6	25.5	62.6	65.0	63.3	70.2	47.2	50.9	37.6^a^	74.4^a^
16	52.5^a^	46.8	2.7	2.8	87.8	82.6	48.7	52.3	61.1	66.8	24.9	21.9	81.8	77.8	73.8	73.1	57.0	54.0	42.8	41.8
17	45.1	46.6	1.8	1.8	80.8	79.7	48.2	46.6	59.1	60.8	29.4	27.7	78.1*^a^*	69.2	72.8	71.5	56.6	55.0	45.7^b^	42.8

a Cochran’s outlier.

b Grubbs’ outlier.

**Table 3.** Interlaboratory study results for fructose

Laboratory no.	Fructose, g/100 g																			
	Bran cereal		Mushroom (closed cap)		Chocolate digestive biscuits		Small dog adult kibble		Infant formula		Cauliflower		Hot chocolate drink mix		Instant pudding mix		Ripe banana		Chicken feed	
	A	E	D	N	B	K	F	O	C	S	I	P	G	H	J	R	L	Q	M	T
1	8.2	8.5	0.2	0.2	12.4	12.2	0.1	0.3	0.0	0.0	11.6	12.0	23.6	22.6	0.1	0.6	24.8	24.5	1.4	1.0
2	8.3	8.4	0.2	0.2	12.9	13.4	0.0	0.0	0.0	0.0	12.0	11.7	22.4	22.3	0.5	0.3	24.9	24.9	1.2	1.3
3	8.9	8.6	0.2	0.3	13.1	13.5	0.1	0.2	0.0	0.0	11.8	12.3	23.0	23.7	0.1	0.4	24.5	24.5	1.2	1.3
4	7.3	6.8	0.2	0.2	11.4	12.1	0.0	0.0	0.0	0.0	10.3	10.5	20.3	21.3	0.7	0.0	23.1	22.4	0.9	1.3
5	8.3	8.4	0.0	0.1	12.5	12.4	0.0	0.0	0.0	0.0	12.1	11.8	23.0	20.1	0.2	0.2	24.1	24.4	1.1	1.2
6	8.3	8.3	0.3	0.2	12.8	12.9	0.3	0.2	0.0	0.0	11.6	11.8	24.8	21.8	0.5	0.6	24.7	24.9	1.3	1.3
7	8.6	8.7	0.3	0.3	13.2	13.3	0.2	0.3	0.0	0.0	12.0	12.6	20.0	19.1	0.5	0.6	26.0	25.2	1.4	1.5
8	7.6	7.6	0.2	0.2	11.8	12.3	0.0	0.2	0.0	0.0	11.0	11.9	24.8	22.6	0.1	0.2	24.6	23.4	1.2	1.3
9	8.2	8.3	0.3	0.4	12.7	12.7	0.0	0.0	0.0	0.0	10.4	10.8	20.5	21.9	0.0	0.0	24.9	24.7	1.3	1.2
10	8.5	8.3	0.2	0.2	12.9	12.9	0.0	0.5	0.0	0.0	11.6	11.7	22.5	21.5	0.3	0.4	24.7	24.8	1.2	1.2
11	8.4	7.6	0.2	0.3	13.1	13.1	0.0	0.2	0.7	0.0	12.6	11.5	17.4	20.2	1.1	0.1	24.9	24.4	0.9	1.2
12	9.0	8.9	0.2	0.1	13.7	13.8	0.6	0.0	0.0	0.0	12.5	12.0	25.4	24.6	0.7	0.2	24.6	24.8	1.6	1.7
13	8.0	8.3	0.2	0.0	12.5	13.0	2.3	0.4	0.0	0.0	11.2	11.6	23.1	23.1	0.2	0.6	23.8	24.5	1.3	1.3
14	9.1	8.9	0.4	0.3	13.8	13.5	0.4	0.4	0.0	0.0	12.3	12.0	24.6	25.4	0.5	0.4	25.4	25.3	1.3	1.2
15	7.9	7.2	0.2	0.2	12.3	10.3	0.4	0.2	0.0	0.0	9.4	10.4	16.6	19.0	0.0	1.2	20.3	21.7	0.8	36.3*^a^*
16	9.7	8.8	1.5	0.7	14.5	13.3	0.5	0.5	0.0	3.6	9.8	12.6	24.3	22.8	0.8	0.5	25.6	25.0	1.5	0.0
17	8.3	9.1	1.7	1.5	13.5	11.3	0.6	0.0	0.0	0.0	13.1	12.2	23.7	17.1	1.0	0.8	25.9	25.0	2.1	1.4
Mean, g/100 g	8.33		0.35		12.80		0.26		0.13		11.61		22.03		0.42		24.45		1.25	

a Analytical error.

**Table 4.** Interlaboratory study results for glucose

Laboratory no.	Glucose, g/100 g																			
	Bran cereal		Mushroom (closed cap)		Chocolate digestive biscuits		Small dog adult kibble		Infant formula		Cauliflower		Hot chocolate drink mix		Instant pudding mix		Ripe banana		Chicken feed	
	A	E	D	N	B	K	F	O	C	S	I	P	G	H	J	R	L	Q	M	T
1	36.7	38.0	1.7	1.6	64.1	63.5	47.8	45.5	29.2	28.4	15.3	15.6	47.6	47.0	64.4	62.7	29.8	30.0	40.9	40.4
2	38.2	37.5	1.8	1.8	66.6	66.9	46.9	46.9	30.4	29.0	15.5	15.7	46.1	46.4	62.6	64.2	30.9	30.3	40.9	39.7
3	40.7	40.3	2.0	1.8	70.6	71.1	52.6	48.9	27.5	30.1	16.7	16.8	49.0	50.0	67.1	65.4	30.3	30.8	42.6	41.6
4	37.0	36.4	1.8	1.8	65.3	64.0	44.2	43.9	27.4	27.0	15.6	15.8	45.3	45.3	62.4	61.2	29.0	28.4	37.8	37.2
5	37.7	38.2	1.9	1.9	67.3	67.1	47.1	47.0	29.5	29.1	16.7	16.6	48.1	47.3	64.6	65.4	30.8	31.5	40.3	40.3
6	37.6	37.1	1.8	1.8	65.7	65.5	46.0	46.6	28.6	28.6	15.6	15.6	46.9	46.4	63.5	62.7	29.1	29.4	40.0	39.3
7	39.3	38.7	2.0	2.0	67.6	69.4	49.0	50.9	30.4	30.2	16.1	16.4	47.2	45.9	62.2	67.0	35.5	32.3	45.1	45.3
8	36.2	36.3	2.3	2.2	63.6	64.8	44.6	45.3	29.2	27.7	15.4	16.5	49.4	48.7	63.2	64.7	29.8	29.4	40.6	40.2
9	38.6	38.5	1.8	1.8	67.7	68.3	47.8	47.5	29.5	29.3	14.2	14.8	46.6	47.2	64.7	64.5	32.0	30.8	41.6	41.4
10	38.0	37.8	2.0	2.1	66.8	66.9	47.6	46.7	29.2	29.0	15.6	15.6	46.6	47.1	62.6	63.3	30.7	31.0	39.4	40.5
11	37.1	34.9	1.8	1.8	62.1	65.5	43.3	44.5	29.3	27.2	16.7	16.1	46.4	46.5	68.7	62.9	34.2	33.6	41.7	41.1
12	40.1	38.6	1.9	1.9	68.0	71.1	47.8	48.2	28.8	28.2	15.6	16.0	48.9	47.5	63.3	64.4	31.0	31.3	41.7	40.5
13	38.1	38.0	1.9	2.1	66.8	68.9	45.5	47.0	29.3	29.4	15.9	15.9	48.9	48.5	66.3	64.0	30.3	30.6	40.5	40.9
14	39.0	39.1	1.8	1.8	68.5	68.9	48.1	47.4	29.9	29.5	16.2	15.8	48.2	48.2	65.3	65.0	25.4	32.0	41.0	41.2
15	36.6	36.0	2.0	2.1	65.6	57.9	45.9	42.3	26.5	33.3	14.1	14.7	41.0	41.2	56.6	61.2	26.8	28.6	36.7	38.2
16	40.7	38.1	0.9	2.0	71.5	66.5	48.5	48.4	31.1	30.0	10.8	12.2	51.0	49.1	65.6	66.9	30.4	30.0	37.7	29.7
17	36.9	37.6	1.8	1.8	65.4	66.2	47.2	47.7	29.1	30.6	16.5	15.4	48.0	45.8	64.2	63.0	31.1	29.6	42.8	41.0
Mean, g/100 g	37.93		1.87		66.64		46.90		29.16		15.53		47.16		63.99		30.49		40.29	

**Table 5.** Interlaboratory study results for galactose

Laboratory no.	Galactose, g/100 g																			
	Bran cereal		Mushroom (closed cap)		Chocolate digestive biscuits		Small dog adult kibble		Infant formula		Cauliflower		Hot chocolate drink mix		Instant pudding mix		Ripe banana		Chicken feed	
	A	E	D	N	B	K	F	O	C	S	I	P	G	H	J	R	L	Q	M	T
1	0.0	0.0	0.0	0.0	2.0	2.1	0.0	0.1	29.3	28.7	0.0	0.2	5.5	5.8	7.5	6.7	0.0	0.0	0.1	0.2
2	0.0	0.0	0.0	0.0	2.3	2.2	0.4	0.0	30.8	29.3	0.2	0.1	5.7	5.8	7.3	7.9	0.0	0.0	0.0	0.1
3	0.0	0.0	0.0	0.1	2.2	2.2	−0.8	0.3	27.0	30.5	0.0	0.3	5.7	6.3	8.1	7.9	0.1	0.2	0.2	0.2
4	−0.1	0.0	0.0	0.0	1.8	2.1	−0.2	−0.8	27.1	26.9	−0.1	−0.8	5.3	5.0	7.3	6.7	−0.2	−0.8	−0.2	−0.8
5	0.0	0.0	0.0	0.1	1.6	1.8	−0.4	−0.7	28.7	29.1	−0.4	−0.5	5.3	5.6	7.2	7.4	−0.2	−0.5	−0.6	−0.2
6	0.0	0.0	0.0	0.0	2.0	2.1	0.1	0.0	28.9	28.8	0.0	0.1	5.2	5.9	7.4	7.0	0.0	0.0	0.0	−0.1
7	0.0	0.1	0.0	0.0	2.2	2.2	0.2	−0.1	30.0	30.1	0.1	0.7	6.3	6.3	7.4	8.0	0.2	−0.2	−0.1	−0.1
8	0.1	0.3	0.0	0.0	2.3	1.9	0.0	−0.1	29.1	27.2	0.2	−0.1	5.8	5.9	7.6	7.4	0.2	−0.2	0.0	0.2
9	−0.1	0.0	0.0	0.1	2.1	2.3	0.0	0.0	30.0	29.5	0.2	0.2	6.0	5.9	7.9	7.6	0.0	0.0	0.1	0.2
10	0.1	0.1	0.1	0.1	2.2	2.1	0.1	0.0	29.7	29.3	0.2	0.0	5.7	6.0	7.5	7.4	0.0	0.0	0.0	0.0
11	−1.2	0.3	0.0	0.0	1.0	2.3	0.1	−0.3	29.1	27.4	0.3	−0.4	6.7	6.4	8.7	7.5	0.0	−0.2	−0.2	−0.2
12	−0.2	−0.3	0.0	0.0	2.7	2.2	0.2	−0.2	30.1	28.8	−0.2	0.0	5.6	5.8	7.8	7.6	0.2	−0.5	0.1	−0.4
13	0.0	0.0	0.0	0.0	2.2	2.1	0.0	0.0	29.5	29.4	0.0	0.1	5.7	5.8	8.1	7.8	0.1	0.0	0.0	0.0
14	0.0	0.0	0.1	0.0	2.3	2.4	0.0	0.0	30.1	29.9	0.1	0.1	5.7	5.4	7.8	7.8	0.0	0.0	0.0	0.0
15	0.0	0.1	0.1	0.1	2.3	2.0	0.2	0.0	31.0	34.3	0.0	0.4	4.9	4.8	6.7	7.7	0.0	0.6	0.0	0.1
16	2.0	0.0	0.3	0.2	1.8	2.9	−0.3	4.4	30.3	33.3	0.4	−0.4	6.5	6.0	7.4	7.8	1.0	−1.0	3.6	−0.3
17	0.0	0.0	0.0	0.0	2.0	2.1	0.4	1.0	30.5	30.4	0.0	0.1	6.3	6.2	7.6	7.5	−0.3	0.2	0.8	0.3
Mean, g/100 g	0.04		0.04		2.12		0.11		29.53		0.03		5.79		7.56		-0.04		0.09	

**Table 6.** Statistical evaluation of interlaboratory study results for available carbohydrates (AVCHO method) including all experimental values (i.e., without any statistical outliers removed)

	Bran cereal	Mushroom (closed cap)	Chocolate digestive biscuits	Small-dog adult kibble	Infant formula	Cauliflower	Hot chocolate drink mix	Instant pudding mix	Ripe banana	Chicken feed
Samples	A & E	D & N	B & K	F & O	C & S	I & P	G & H	J & R	L & Q	M & T
No. of laboratories	17	17	17	17	17	17	17	17	17	17
No. of replicates	34	34	34	34	34	34	34	34	34	34
Mean, g/100 g	46.20	2.15	81.55	47.28	58.38	27.21	74.99	71.92	55.09	43.03
S_r_, g/100 g	1.15	0.08	2.25	1.29	3.26	0.94	2.13	2.23	1.28	6.35
S_R_, g/100 g	1.99	0.25	3.53	2.31	3.26	1.68	4.20	2.50	2.58	6.35
RSD_r_, %	2.49	3.91	2.77	2.73	5.59	3.46	2.84	3.10	2.32	14.76
RSD_R_, %	4.30	11.58	4.32	4.88	5.59	6.19	5.61	3.47	4.68	14.76

**Table 7.** Combined standard and expanded uncertainties of the available carbohydrates (AVCHO) method

Sample^a^	Mean available carbohydrates, g/100 g	Standard uncertainty *U_c_* (C_Available Carbohydrates_) (%)	Expanded uncertainty^b^*U* (C_Available Carbohydrates_) (%)
A & E	46.01	1.67	3.34
D & N	2.15	0.25	0.50
B & K	81.89	2.94	5.88
F & O	47.28	2.31	4.61
C & S	58.01	2.04	4.07
I & P	27.21	1.68	3.37
G & H	74.89	4.24	8.48
J & R	71.92	2.50	4.99
L & Q	55.09	2.58	5.16
M & T	42.20	1.81	3.62

a Samples: A&E = bran cereal; D&N = mushrooms (closed cap); B&K = chocolate digestive biscuits; F&O = small dog adult kibble. C&S = infant formula; I&P = cauliflower; G&H = hot chocolate drink mix; J&R = instant pudding mix; L&Q = ripe banana. M&T = chicken feed.

b Expanded uncertainty calculated using a coverage factor of 2, which gives a level of confidence of approximately 95%.

Available carbohydrates levels ranged from 1.8% to 87.8% (Table 2). Raw data for total available carbohydrates from the collaborative study are shown in Table 2, with Cochran’s and Grubbs’ outliers indicated. Data for the individual sugars are shown in Tables 2–5. The statistical results for available carbohydrates, before and after removal of the outliers, are shown in Tables 6 and **2020.07**, respectively. The s_r_ for total available carbohydrates ranged from 0.08 to 2.23 g/100 g, and s_R_ ranged from 0.25 to 4.24 g/100 g. RSD_R_ ranged from 3.47 to 11.58%, with 9 of the 10 samples having RSD_R_ of <6.19%. Measurement uncertainty (*U*) ranged from 0.50 to 8.48% (Table 7). Repeatability, reproducibility, and measurement uncertainty values were within the range of performance characteristics typically found for similar methods or similar analytical formats [e.g., methods for measurement of dietary fiber (16)].

Mushrooms, with ∼2% available carbohydrates, were included in the study to highlight the need for larger aliquots for the analysis or lesser dilution when analyzing samples with <10% available carbohydrates. In-house studies showed that in the measurement of the available carbohydrates in the mushroom preparations, the analysis of 0.5 or 1.0 mL of a 26-fold diluted incubation solution, or of 0.1 or 0.2 mL of a 6-fold dilution of the incubation solution, gave similar analytical values.

### Statistical Treatment

Collaborating laboratory data were evaluated statistically according to AOAC INTERNATIONAL protocols using AOAC-supplied software. Of the 10 valid pairs of assay results reported from 17 laboratories for available carbohydrates, laboratories 1 through to 14 had no statistical outliers, and laboratories 15, 16, and 17 had eight statistical outliers with a total of total statistical outlier pairs.

The raw data and statistically paired data from the blind duplicate results for available carbohydrates reported by the collaborating laboratories are shown in Tables 2–5, 6, and **2020.07**, respectively. Outliers and the reason for outlier removal are indicated and footnoted in Table 2.

Measurement uncertainty of the Available Carbohydrates (AVCHO) procedure was assessed and calculated in accordance with the guidelines specified by Eurachem CITAC Guide CG4 (QUAM : 2012.P1). The use of the reproducibility standard deviation (s_R_) derived from a collaborative study as a measure of the combined standard uncertainty of the method is appropriate on the basis that: this is an empirical method whereby the measurement is dependent on the method used, there is no available certified reference material (CRM), the method has been subject to a collaborative study whereby laboratory collaborators performed all stages of the method.

The method was applied to collaborative study using 10 samples matrixes (20 homogeneous test samples as 10 blind duplicates) analyzed by 17 collaborators.

Expanded uncertainty (*U*) is calculated using a coverage factor (*k*) of 2 on the basis that all mean results have a degrees of freedom greater than 25. The coverage factor (*k*) of 2 provides a level of confidence of approximately 95%.

The total available carbohydrates value of a given sample is the sum total of the individual monosaccharides fructose, glucose, and galactose from each of the carbohydrates analyzed. Raw data for each of the individual monosaccharides (fructose, glucose, and galactose) reported by the collaborating laboratories are shown in Tables 3–5, respectively. Extremely high or low RSD_R_ values for the individual monosaccharides (i.e., outside of the range 0–14.7%) can be explained either by the fact that the measurement values were close to zero, and below the limit of detection for the given assay, or by an obvious single outlier. Outside of this reasoning the RSD_R_ for any of the monosaccharides across the samples ranged from 3.4 to 14.7%.

Some of the values reported for fructose and galactose were negative in value but extremely close to zero. The negative values are a result of a very slightly negative absorbance difference between two absorbance readings of the same reaction tube at different stages and is an artifact of the assay. Since it is not physically possible for a sample to have a negative content of any of the monosaccharides these negative values should theoretically be taken as being zero. The contribution by any of the negative values to the total carbohydrates measurement was negligible, versus using a corrected value of zero; therefore, none of the values were corrected, and raw data were used for the calculation of total available carbohydrates. Correcting negative values to zero would also complicate the results analysis process.

In addition, any variability associated with the individual monosaccharide measurements contributes to repeatability, reproducibility, and measurement uncertainty for the whole of the total available carbohydrates method. As previously mentioned, each of these statistical parameters for the whole total available carbohydrates method was well within the range of performance typically expected for similar methods or analytical formats.

## Discussion and Collaborator Comments

The pretrial practice sample analyses identified sections of the method description that needed improvement and provided insights into the analysis of samples containing resistant starch, including RS_4_ wheat starch and cooked potato. In these analyses, no specific problems were identified by the collaborators; however, several suggestions were received that helped improve the clarity of the method. Collaborators reported the procedures they used in the initial incubation step with PAA/AMG. These included the recommended procedures of mixing or stirring in a temperature-controlled water bath, intermittent regular mixing followed by incubation in a water bath at 37°C, or mixing and incubation in a temperature-controlled oven. Each of the methods performed adequately, but intermittent, manual mixing is very labor-intensive and not recommended. Various questions concerning the exact conditions required and flexibility in performing the cuvette incubations were asked, including temperature control and the method for mixing the cuvette contents. Collaborators were advised that the various reactions would be completed if the cuvettes were incubated for the stated time within the temperature range of 20–30°C. The preferred method for mixing cuvette contents is by covering the cuvette with Parafilm^®^ and inverting the cuvette a few times.

When analyzing the practice samples, helpful comments were made by several collaborators, and these suggestions were incorporated into the method text before the full MLV was performed. Comments from collaborators, particularly those related to section sections **B**(**e**)*(1)* and *(2)* (preparation of PAA/AMG for use in incubations) and section **G**(**a**) (procedure for incubation of sample with either soluble PAA/AMG, of an ammonium sulphate suspension of these two enzymes) were adopted, and the procedures were clarified as a result. In tabulating the results from the MLV, data for some samples were clearly inconsistent with that from other laboratories, so to identify the reasons for this inconsistency, the collaborators were contacted. The data submitted by Laboratory 17 were, in general, consistent with that obtained from other laboratories except for data for samples D and N. The available carbohydrates values reported were 7.7% for sample D and 7.4% for sample N. The collaborator was contacted and said, “Finally we found that the lab technician had a mistake on pipetting among samples.” Consequently, we accepted a second set of data for these samples (*see*Tables 2–5). Laboratory 15 reported a very high value for the available carbohydrates value of sample T. This is due to the very high value obtained for the fructose content of this sample (36.3%), compared to the value obtained for the replicate sample (sample M; 0.8%) and the average value obtained for all laboratories for this sample (1.2%). The collaborator stated they would rerun the sample to identify the cause of the problem. This rerun was not performed. Other variations in values for the replicate samples, as reported in Table 1, are most probably due to pipetting errors.

## Conclusions

Based on the results obtained from this study, it is recommended that the AVCHO method **2020.07** for the measurement of available carbohydrates be adopted as Final Action Official method.
